# Publisher Correction: Small molecule inhibition of cGAS reduces interferon expression in primary macrophages from autoimmune mice

**DOI:** 10.1038/s41467-017-01770-3

**Published:** 2017-11-23

**Authors:** Jessica Vincent, Carolina Adura, Pu Gao, Antonio Luz, Lodoe Lama, Yasutomi Asano, Rei Okamoto, Toshihiro Imaeda, Jumpei Aida, Katherine Rothamel, Tasos Gogakos, Joshua Steinberg, Seth Reasoner, Kazuyoshi Aso, Thomas Tuschl, Dinshaw J. Patel, J. Fraser Glickman, Manuel Ascano

**Affiliations:** 1 Vanderbilt University School of Medicine, Nashville, TN 37027 USA; 2 The Rockefeller University, New York, NY 10065 USA; 3 Structural Biology Program, Memorial Sloan-Kettering Cancer Center, New York, NY 10065 USA; 4 Key Laboratory of Infection and Immunity, CAS Center for Excellence in Biomacromolecules, Institute of Biophysics, Chinese Academy of Sciences, Beijing, 100101 China; 5 Howard Hughes Medical Institute Laboratory for RNA Molecular Biology, New York, NY 10065 USA; 6 Tri-Institutional Therapeutics Discovery Institute, New York, NY 10021 USA; 7 Present Address: Regeneron Pharmaceuticals Incorporated, Tarrytown, NY 10591 USA

*Nature Communications* **8**:750 doi:10.1038/s41467-017-00833-9; Article published online 29 September 2017

The previously published version of this Article contained errors in Fig. 6. In panel h the units of the *x* axis were incorrectly given as mM and should have been given as µM. Also, the IC_50_s for RU.365, RU.332 and RU.521 within panel h were incorrectly given as mM and should have been given as µM. These errors have been corrected in both the PDF and HTML versions of the Article.

