# Supplementary material for: Small molecule inhibition of cGAS reduces interferon expression in primary macrophages from autoimmune mice
Source: Nat Commun. 2017 Sep 29;8:750. doi: 10.1038/s41467-017-00833-9 (PMC5622107; doi:10.1038/s41467-017-00833-9)
Supplement: Supplementary file 1 — Supplementary Information [file 41467_2017_833_MOESM1_ESM.pdf]

Description of Supplementary Files

File name: Supplementary Information
Description: Supplementary figures and supplementary tables.

File name: Peer review file

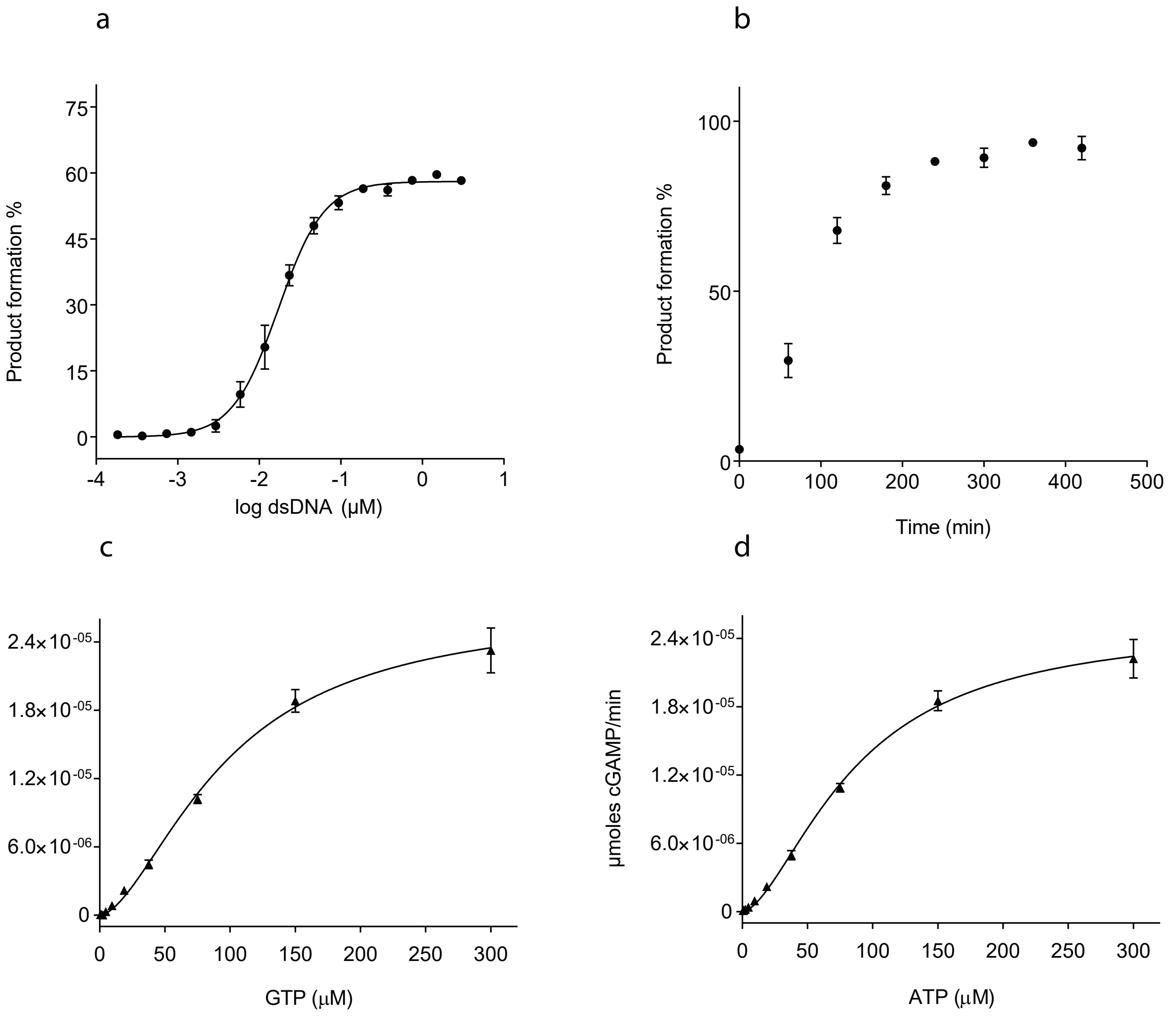

Enzymatic parameters					
ATP			GTP		
V_{max}^{app} (μmoles cGAMP/min	K_m^{app} (μM)	k_{cat} (min^{-1})	V_{max}^{app} (μmoles cGAMP/min	K_m^{app} (μM)	k_{cat} (min^{-1})
$2.5x10^{-5}$	86.0	23	$2.7\ x10^{-5}$	96.4	23

Supplementary Fig. 1. Optimization and development of in vitro cGAS assay for high-throughput implementation. (a) Activity of mouse cGAS determined by the RapidFire Mass Spectrometry system (RF-MS). The 50% effective concentration (EC50) of dsDNA calculated from Fig. 1b by fitting the area under the curve of the cGAMP molecular ion signal to a sigmoidal dose-response. (b) Formation of cGAMP monitored over time Using 0.3 μM dsDNA. cGAS kinetics using RF-MSS. Substrate dependence of activity of mouse cGAS for ATP (c) and GTP (d). The inset table shows k_{cat} and K_m obtained for both substrates. The calculations were based on 3 different experiments and the curves are non-linear fits generated by GraphPad Prism.

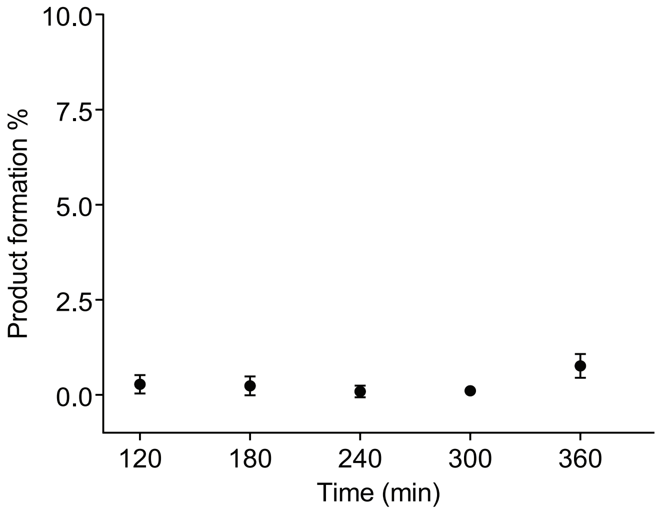

Supplementary Fig 2. Time course of h-cGAS using the same conditions used for the m-cGAS screen.

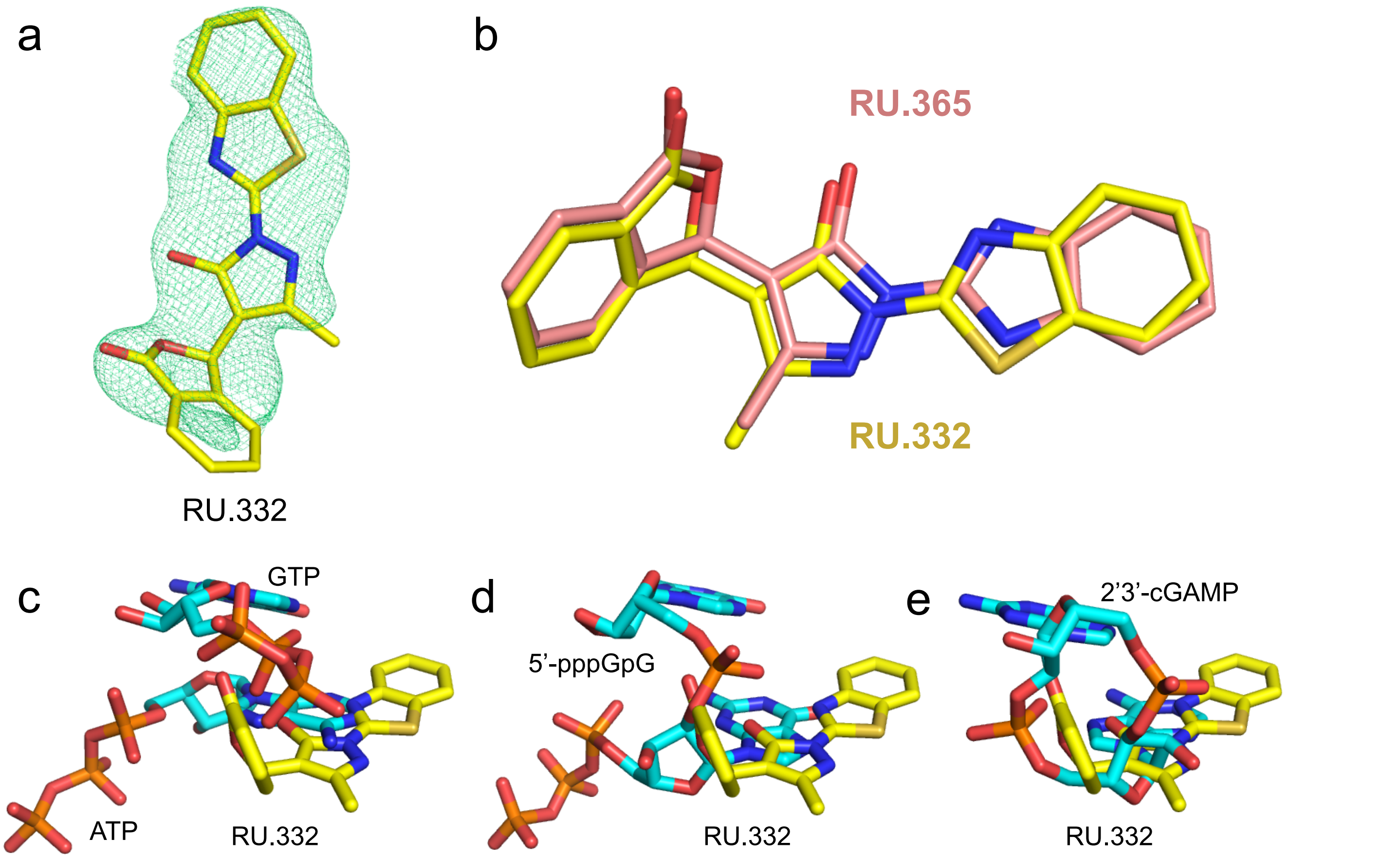

Supplementary Fig. 3. A structural comparison of RU.365 with its analog RU.332. (**a**) Omit Fo-Fc electron density (green mesh) of RU.332 contoured at 3σ. (**b**) Superposition of structures of bound RU.365 (salmon) and RU.332 (yellow). Superposition of structures of bound RU.332 (**c-e**) with those of substrates, intermediate, and product. All the ligands are shown in stick representation. Inhibitors are shown in yellow. Substrates/Intermediate/Product are shown in cyan.

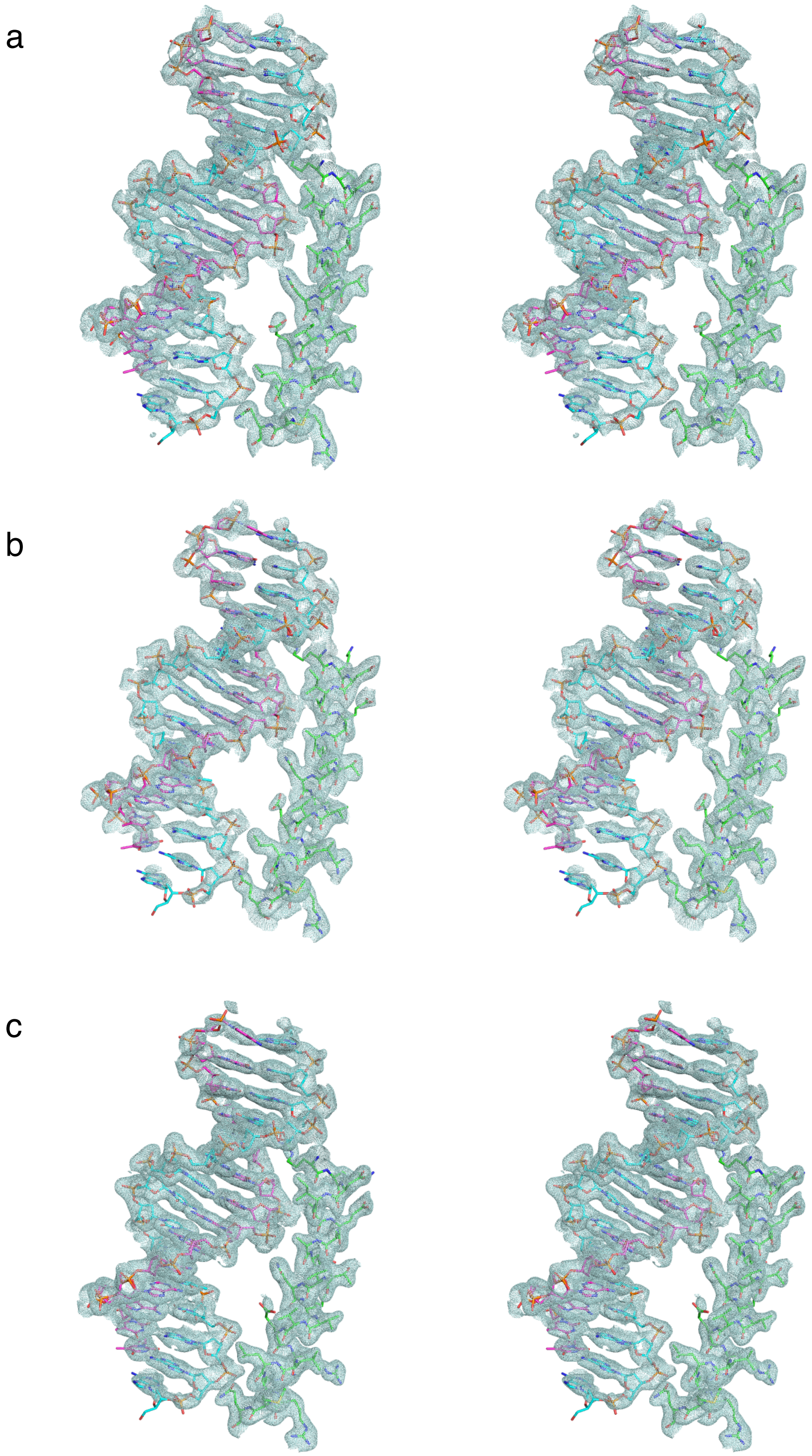

Supplementary Fig. 4. Stereo views of the electron density maps of cGAS ternary complexes. The stereo view of the 2Fo-Fc electron density map (gray) for cGAS (R161-Q183, green) and the bound DNA (cyan and purple) in the structures of cGAS-DNA-RU.365 (**a**), cGAS-DNA-RU.332 (**b**), and cGAS-DNA-RU.521 (**c**). The contour level of the map is 1.0.

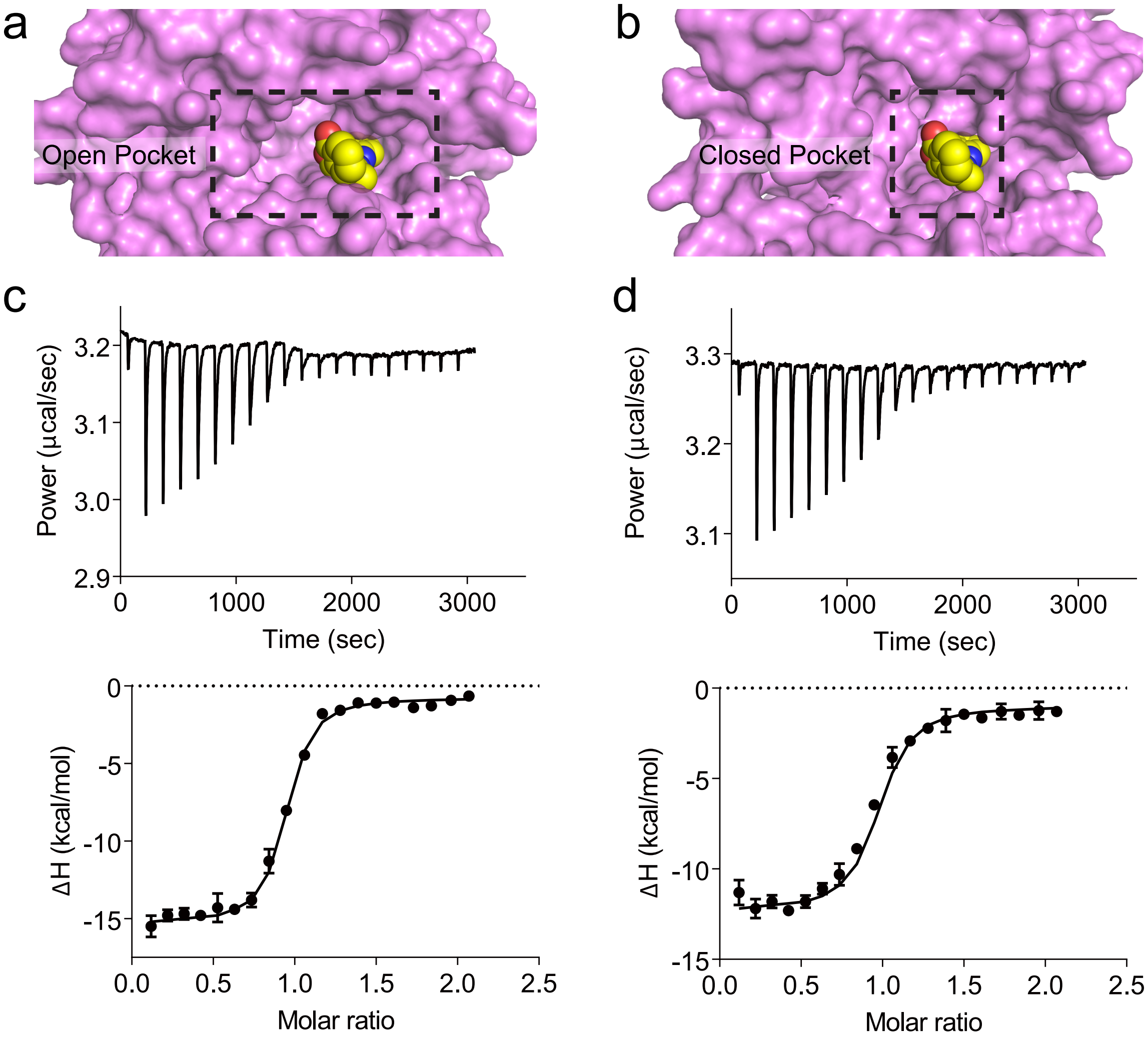

	rM	K_d (nM)	ΔH (cal/mol)	T*ΔS (cal/mol)	ΔG (cal/mol)
cGAS in presence of dsDNA	0.9075	64.09	-14320	-4504	-9813
cGAS in absence dsDNA	0.9384	98.47	-11070	-1511	-9559

Supplementary Fig. 5. Isothermal titration calorimetry binding curves for RU.365 and cGAS in the presence and absence of dsDNA. (**a**) Structure of RU.365 bound to the "open pocket" of cGAS in the dsDNA-bound state. (**b**) Model of RU.365 bound to the "closed pocket" of cGAS in the dsDNA-free state. (**c**) RU.365 titrated into cGAS/dsDNA complex with 1 mM of ATP and (**d**) RU.365 titrated into cGAS/dsDNA complex with1 mM of GTP. The table contains the dissociation constant and thermodynamic parameters.

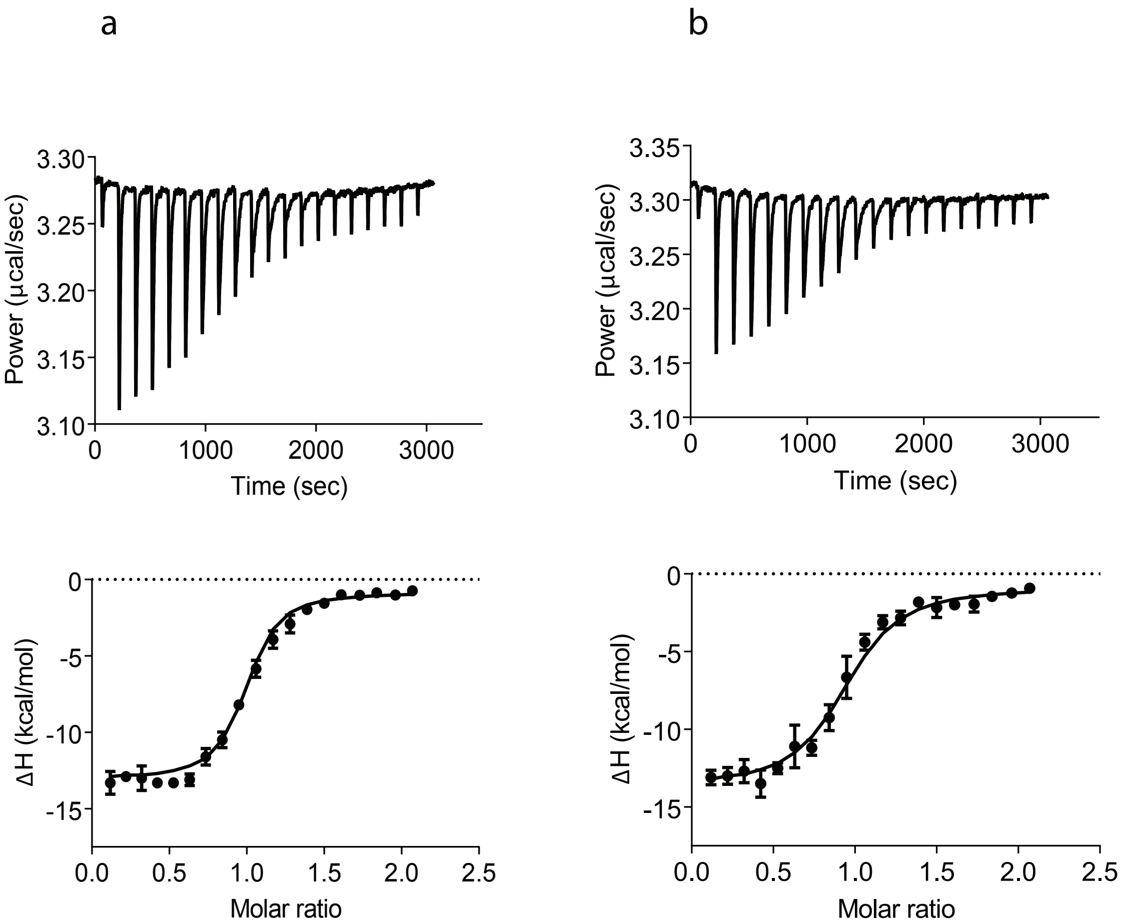

	rM	K_d (nM)	ΔH (cal/mol)	T*ΔS (cal/mol)	ΔG (cal/mol)
cGAS/dsDNA + ATP	0.9605	104.4	-12020	-2493	-9524
cGAS/dsDNA + GTP	0.9257	298.2	-12570	-3668	-8902

Supplementary Fig. 6. Isothermal titration calorimetry binding curves to compare inhibitor binding to cGAS versus its natural substrates. (**a**) RU.365 titration into cGAS/dsDNA complex with 1 mM of ATP and (**b**) RU.365 titration into cGAS/dsDNA complex with 1 mM of GTP. The table contains the dissociation constant and thermodynamic parameters.

a

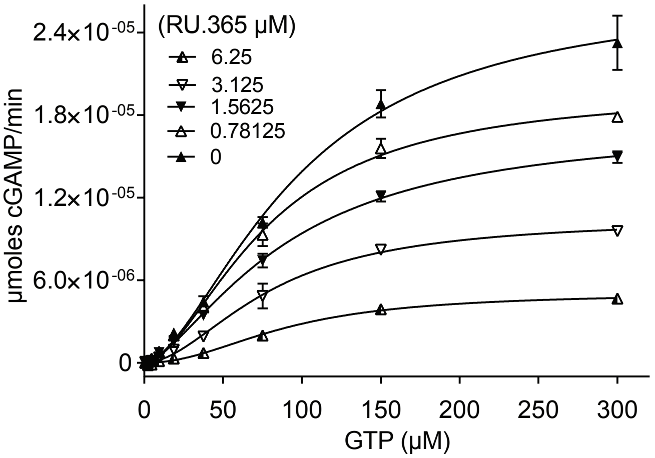

**RU.365** (μM)	6.3	3.2	1.6	0.8	0
V_{max}^{app} (μmoles cGAMP/min)	$5.1x10^{-6}$	$1.1x10^{-5}$	$1.7 x10^{-5}$	$2.0x10^{-5}$	$2.7 x10^{-5}$
K_m^{app} (μM)	89.4	79.7	89.7	77.8	96.4
k_{cat} (min^{-1})	4.3	9.1	14	17	23

b

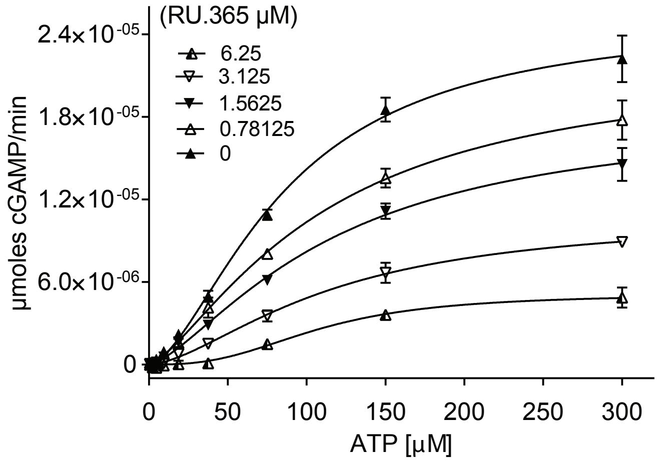

**RU.365** (μM)	6.3	3.2	1.6	0.8	0
V_{max}^{app} (μmoles cGAMP/min)	$5.1x10^{-6}$	$1.1x10^{-5}$	$1.8 x10^{-5}$	$2.2x10^{-5}$	$2.5 x10^{-5}$
K_m^{app} (μM)	107.3	113.4	115.5	109.1	86.0
k_{cat} (min^{-1})	4.3	9.1	14	17	23

Supplementary Fig 7. Kinetics of m-cGAS in the presence and absence of RU.365 (**a**) Progress curves of m-cGAS varying GTP at fixed concentration of ATP, table on the right shows the values for K_m^{app}, V_{max} and k_{cat}. (**b**) Progression curves of m-cGAS varying ATP at fixed concentration of GTP, table on the right shows the values for K_m^{app}, V_{max} and k_{cat} determined by the RapidFire Mass Spectrometry system (RF-MS).

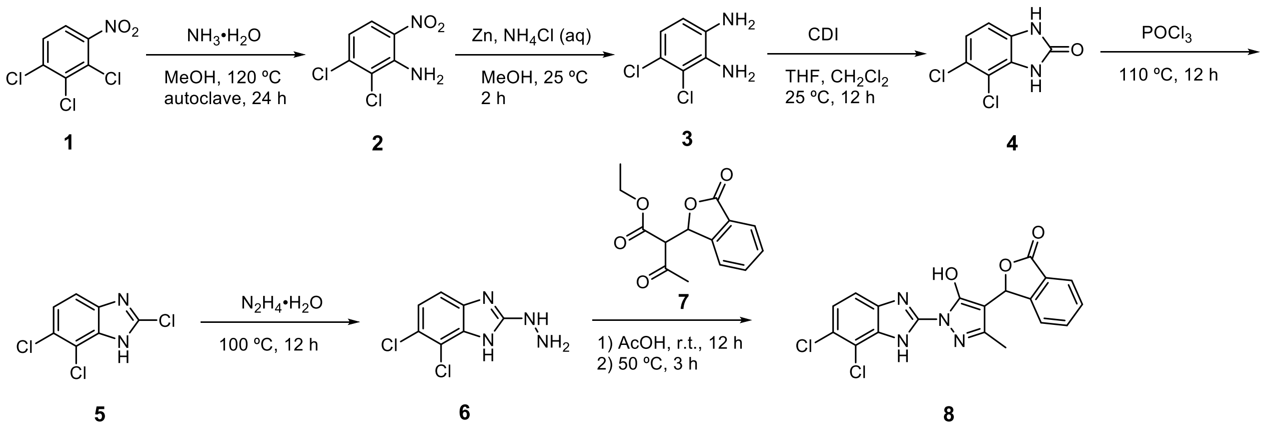

Supplementary Fig. 8. Synthesis of RU.521. Chemical synthesis scheme depicting the synthesis of RU320521 (RU.521).

Supplementary Table 1. cGAS high-throughput small molecule screening data summary

Category	Parameter	Description
Assay	Type of assay	Purified enzyme assay
	Target	Mouse Cyclic GMP-AMP Synthase (m-cGAS)
	Primary measurement	Mass spectrometry
	Key reagents	m-cGAS, ATP, GTP, dsDNA
	Assay protocol	20 µL final volume: Buffer of the reaction containing 20 mM Tris-HCl pH 7.4, 150 mM NaCl, 5mM $MgCl_2$, 1mM DTT and 0.01% Tween-20. Samples contains buffer of the reaction, 300 µM ATP and GTP, 300 nM dsDNA and 60 nM cGAS incubated for 120 min at room temperature. The reaction was stopped with 65µL formic acid 0.5%. Samples were analyzed in RF-MSS. Order of addition: 5 µL of reaction buffer, 0.05 µL compounds, 10 µL of a mix of ATP, GTP and dsDNA (columns 1-23), 10 µL of a reaction containing ATP, GTP (column 24), 5 µL solution containing cGAS (full plate). The plates were spun down for 30 seconds at 180 x g. After 120 min at room temperatures the reaction was stopped with formic acid 0.5%.
	Additional comment	Samples were analyzed using RF-MSS. The load/wash solvent was water containing 5 mM ammonium acetate, pH 10 (solvent A). The elution solvent was 50% water, 25% acetone and 25 % acetonitrile containing 5 mM ammonium acetate, pH 10 (solvent B). Approximately 35 µL of samples were injected to a Graphitic carbon cartridge (Type D). Once the sample was loaded into the cartridge a wash step of 4000 ms at 1.5 mL/min was performed to remove buffer salts. To elute the analytes of interest (ATP, GTP and cGAMP) an

		elution step of 5000 ms with buffer B was performed at a flow rate of 1.5 mL/min. The cartridge was after re-equilibrated with solvent A for 5000 ms at a flow rate of 1.5 mL/min.
Library	Library size	123,306 pure compounds
	Library composition	Low molecular weight compounds were a random subset from the Rockefeller University Compound Library of 283,000 compounds
	Source	ChemDiv, Spectrum, Prestwick, Enamine, Pharmakon-900, LifeChem, Specs, Chiral Center Diversity Library, NIH Clinical Collection, Tocris, Biofocus, HTSRC Clinical Collection, Cerep, Chembridge, AMRI, Analyticon, ChemX.
	Additional comments	Library description www.rockefeller.edu/htsrc/
Screen	Format	384-well plate
	Concentration tested	12.5 µM; 0.25% DMSO
	Plate controls	DMSO (negative control), no dsDNA (positive control)
	Reagent/compound dispensing system	MultiDrop Combi with RapidStack (Thermo Scientific) for reagents; Janus Automated Workstation with Nanohead (Perkin-Elmer) for compounds.
	Detection instrument and	RapidFire Mass Spectrometry System (Agilent);

	software	Agilent Mass Hunter WorkStation software. RapidFire Integrator software.
	Assay Validation /QC	Hit-compounds were re picked from the original 5 mM source and retested at 10 serial diluted concentrations; in triplicate and a concentration response curved fitted to a 4 parameter hyperbolic using CDD software. HPLC performed to all hits compounds to detect purity.
	Correction factors	none
	Normalization data	The % inhibition is calculates as follows: % inhibition = 100 x (sample - average negative control)/(average positive control - average negative control)]. The quality of the screen was assessed by Z' factor calculated as follows: Z'=1-[3*(standard deviation positive control + standard deviation negative control)/(average positive control - average negative control)]. Catalytic rates expressed as percent product formation were calculated using the AUC of cGAMP and using the AUC's of ATP and GTP to normalize for variations in the capacity of the solid phase extraction column during a screening run, as shown in the following formula: product formation (%) = [(AUC_{cGAMP} x 100) / (AUC_{cGAMP} + ½ AUC_{ATP} + ½ AUC_{GTP})], where AUC: area under the curve.<br>To determine the percentage of inhibition of m-cGAS the data was normalized against the positive control (column 24) and negative control
	Additional comments	
Post-HTS analysis	Hit criteria	Normalized percentage of inhibition ≥60%
	Hit rate	0.19%

	Additional assays	Isothermal titration calorimetry (ITC)
	Confirmation of hit purity and structure	LC-MS; powders re-ordered and independently synthesized and re-tested in concentration – response curves
	Additional comments	All hits compounds reported were at least 85% pure by LC-MS.

Supplementary Table 2. 49 compounds selected with IC_{50} < 10 µM and HPLC-MS ≥85%

Molecule identifier	CXSMILES	Vendor	% inhibition at 12.5 µM	IC_{50} (µM)	Polarization (mP)	% intercalation at 30 µM
RU100840	O=C1NC2=C(C=CC=C2)C1=C1SC(=NC1=O)N1CCCCC1 |c:5,7,14,t:3|	Enamine	88	0.06	269	-8.9
RU107796	NC1=C(C#N)C(C2=CC=C(C=C2)N(=O)=O)C(C#N)=C(N)S1 |c:1,8,10,t:6,18|	Enamine	62	8.66	266	-7.6
RU114757	ClCC(=O)NC1=NC(=CS1)C1=CNC2=CC=CC=C12 |c:7,16,t:5,11,14,18|	Enamine	72	5.38	271	-10.1
RU116287	FC1=C(C=C2SC(=S)NC2=O)C=CC=C1 |w:3.2,c:1,12,14|	Enamine	63	6.51	271	-9.8
RU1535	OC(=O)COC1=CC(Cl)=C(Cl)C=C1Cl |c:11,t:5,8|	Spectrum	98	0.55	244	3.4
RU161	OC1=C(CC2=C(Cl)C(Cl)=CC(Cl)=C2O)C(Cl)=C(Cl)C=C1Cl |c:1,4,8,11,19,t:16|	Spectrum	93	0.39	108	69.6
RU162145	COC1=C2C(=O)C3=C(C(O)=C4C[C@](O)(C[C@H](O[C@H]5C[C@H](N)[C@@H](O)[C@H](C)O5)C4=C3O)C(=O)CO)C(=O)C2=CC=C1 |c:2,28,39,41,t:6,9|	Sequoia Research Products Ltd.	98	0.58	53	96.5
RU166365	CC1=NN(C2=NC3=CC=CC=C3N2)C(O)=C1C1OC(=O)C2=C1C=CC=C2 |c:8,10,16,23,26,28,t:1,4,6|	ChemBridge	99	0.52	264	-6.7
RU1667	CCOC1=CC=C2N=C3C=C(N)C=CC3=C(N)C2=C1 |c:12,19,t:3,5,7,9,15|	Spectrum	92	1.01	139	54.5
RU169196	CCC1=NN2C(S1)=NC(=O)\C(=C/C1=CC=C(O)C=C1)C2=N |c:7,18,t:2,13,15|	ChemBridge	89	4.25	266	-7.5
RU169248	CCC1=NN2C(S1)=NC(=O)\C(=C/C1=CC=CS1)C2=N |c:7,15,t:2,13|	ChemBridge	97	3.34	262	-5.7

RU171395	CC1=CC=C(C=C1)C(=O)C1=NC2=C(CCCCC2)N1O |c:3,5,t:1,10,12|	ChemBridge	90	0.25	251	-0.3
RU173673	O=C1N=C(NC2=NC=CS2)S\C1=C/C1=CC=CC(=C1)N(=O)=O |c:7,17,19,t:2,5,15|	LifeChem	84	< 0.04	275	-11.7
RU174368	CC1=NN=C(NC(=O)C2=CC=C(O2)S(=O)(=O)NC2=CC=CC(=C2)C(F)(F)F)S1 |c:10,20,22,t:1,3,8,18|	LifeChem	76	7.47	239	5.6
RU185491	CC(C)C1=CC2=C(OC3=C(C=C(C(O)=O)C(N)=N3)C2=O)C=C1 |c:16,22,t:3,5,8,10|	Sequoia Research Products Ltd.	65	9.43	248	1.5
RU187170	NC(=O)N1C(=O)\C(=C(/O)C2=CC=CS2)C2=CC(Cl)=CC=C12 |c:11,18,t:9,15,20|	Tocris	70	6.09	276	-12.4
RU187299	OC1=C(I)C=C(C=C1N(=O)=O)C#N |c:1,4,6|	Enamine	95	0.58	256	-2.8
RU187339	O=C(NC1=NC=C(S1)N(=O)=O)C1=CC=CS1 |c:5,14,t:3,12|	Vitas M Labs	86	6.62	274	-11.2
RU187428	NC(=N)NN=C1C=CC(C=C1)=NNC(N)=S |c:6,9|	Vitas M Labs	79	9.93	164	42.6
RU191669	BrC1=CC=CC=C1N1C=C(N=N1)C(=O)NC1=NN=CS1 |c:3,5,9,11,19,t:1,17|	Enamine	71	9.09	253	-1.0
RU191752	OC1=CC=CN=C1NC(=O)COC1=C2C=CC=CC2=C(Cl)C=C1 |c:3,5,13,15,17,23,t:1,20|	Enamine	95	0.17	276	-12.4
RU198755	FC1=CC=CC2=C1S(=O)(=O)C\C(=C/C1=CC=C(C=C1)C#N)C2=O |c:3,5,16,18,t:1,14|	Enamine	63	6.33	263	-6.2
RU199	COC(=O)C1=C(C)NC(C)=C(C1C1=CC=CC=C1N(=O)=O)C(=O)OC |c:4,9,15,17,t:13|	Spectrum	91	0.80	260	-4.4
RU207450	O=C1CN2C(NC(=O)C3=C2C2=CC=CC=C2S3)=C1C1=CC=C	Enamine	70	9.69	274	-11.2

	C=C1 |c:8,13,15,19,24,26,t:11,22|					
RU207488	CS(=O)(=O)C1=NC=C2N3C(=O)C4=CC=CC=C4NC3=C(C#N)C(=O)C2=N1 |c:13,15,27,t:4,6,11,20|	Enamine	67	4.68	272	-10.2
RU209189	COC(=O)C1=NN2C(N1)=NS(=O)(=O)C1=CC=CC=C21 |c:9,16,t:4,14,18|	Enamine	91	4.44	251	0.0
RU219771	OCC1=CN(N=N1)C1=CC=CC=C1Cl |c:5,10,12,t:2,8|	Enamine	94	1.27	253	-1.3
RU234543	OC[C@H]1CCCN1CC1=CC=C(C=C1)C1=NC(CCN2C=CC=N2)=CC(=O)N1 |c:11,13,22,24,26,t:9,16|	ChemBridge	89	6.76	270	-9.3
RU24114	COC1=C2C(=O)C3=C(C(O)=C4C[C@](O)(C[C@H](O[C@H]5C[C@H](N)[C@H](O)[C@H](C)O5)C4=C3O)C(=O)CO)C(=O)C2=CC=C1 |c:2,28,39,41,t:6,9|	Sequoia Research Products Ltd.	96	0.82	53	96.8
RU24146	COC1=CC=CC2=C1C(=O)C1=C(O)C3=C(C[C@](O)(C[C@@H]3OC3CC(N)C(O)C(C)O3)C(C)=O)C(O)=C1C2=O |c:4,6,11,37,t:2,14|	LightBiologicals	93	0.69	55	95.7
RU297	OC1=CC=C(C=C1N(=O)=O)[As](O)(O)=O |c:3,5,t:1|	Spectrum	96	1.37	271	-9.8
RU316	CCN(CC)CCCC(C)NC1=C2C=C(OC)C=CC2=NC2=CC(Cl)=CC=C12 |c:11,17,20,25,t:13,22,27|	Spectrum	68	6.22	140	54.0
RU3433	ClC1=C(SC2=CC=CC=C12)C1=NNC(=S)O1 |c:6,t:1,4,8,12|	Enamine	92	6.12	288	-18.4
RU431	NC1=C2C=CC=CC2=NC2=CC=CC=C12 |c:1,3,5,8,12,t:10,14|	Spectrum	78	2.83	145	51.5
RU4893	CCOC(=O)C1=NNC2=C1C(=O)N(CC)C2	ChemDiv	62	6.42	254	-1.4

	=O |c:8,t:5|					
RU5004	O=C1NN2C(=O)C3=CC=CC=C3N=C2C2=CC=CC=C12 |c:8,10,13,18,t:6,16,20|	ChemDiv	90	6.49	255	-2.1
RU5512	OC(=O)C1CSC(N1)C1=CC(Br)=C(O)C(Br)=C1 |c:16,t:9,12|	ChemDiv	87	3.00	264	-6.3
RU627	OCCNCCNC1=CC=C(NCCNCCO)C2=C1C(=O)C1=C(O)C=CC(O)=C1C2=O |c:18,23,26,29,t:7,9|	LightBiologicals	94	0.72	41	102.5
RU646	OC[C@H]1O[C@H](OC2=CC=CC3=C2C(=O)C2=C(O)C=C(C=C2[C@@H]3[C@H]2C3=CC=CC(O[C@H]4O[C@H](CO)[C@@H](O)[C@H](O)[C@H]4O)=C3C(=O)C3=C(O)C=C(C=C23)C(O)=O)C(O)=O)[C@H](O)[C@@H](O)[C@@H]1O |c:8,10,15,18,20,28,43,48,51,t:6,26,53|	Spectrum	82	8.76	278	-13.5
RU662	CC[N+]1=C(C2=CC=CC=C2)C2=CC(N)=CC=C2C2=CC=C(N)C=C12 |c:2,6,8,14,16,t:4,11,19,21,24|	Spectrum	97	0.43	42	101.9
RU6753	CC1=NN2C(=N1)N=C(C)C(Br)=C2O |c:4,11,t:1,7|	ChemDiv	69	7.99	252	-0.5
RU803	OC1=CC=C(C=C1N(=O)=O)N(=O)=O |c:3,5,t:1|	Spectrum	96	0.87	260	-4.7
RU83653	COC1=CC=CC2=C1C(=O)C1=C(O)C3=C(C[C@](O)(C[C@@H]3O[C@H]3C[C@H](N)[C@H](O)[C@H](C)O3)C(C)=O)C(O)=C1C2=O |c:4,6,11,37,t:2,14|	Prestwick	91	1.81	50	98.0
RU84314	C[C@@H]1O[C@H](C[C@H](N)[C@@H]1O)O[C@H]1C[C@@](O)(CC2=C(O)C3=C(C(O)=C12)C(=O)C1=CC=CC=C1C3=O)C(C)=O |c:16,19,30,32,t:22,2	Sequoia Research Products Ltd.	90	1.45	72	87.2

	8|					
RU869	CCN(CC)CCNC1=CC=C(CO)C2=C1C(=O)C1=CC=CC=C1S2 |c:14,21,23,t:8,10,19|	Spectrum	66	6.80	85	81.2
RU871	C[N+]1=CC2=C3OCOC3=CC=C2C2=C1C1=CC3=C(OCO3)C=C1C=C2 |c:3,9,11,14,25,28,t:1,17,19|	Prestwick	85	3.37	56	95.0
RU891	CC[C@@]1(O)C[C@H](O[C@H]2C[C@@H]([C@H](O)[C@H](C)O2)N(C)C)C2=C(O)C3=C(C=C2[C@H]1C(=O)OC)C(=O)C1=CC=CC(O)=C1C3=O |c:19,22,24,37,40,t:35|	Spectrum	98	1.42	65	90.6
RU93687	NC(=O)C1=C(NC(=O)CC#N)SC2=C1CCCC2 |c:3,12|	Enamine	91	9.11	263	-5.8
RU94102	CC(C)OC(=O)C(=CC1=CC=C(O1)N(=O)=O)C#N |c:10,t:8|	Enamine	64	8.14	267	-8.1

Supplementary Table 3. Structure activity relationship of analogs with potency of m-cGAS inhibition higher than RU166365

Core Structure

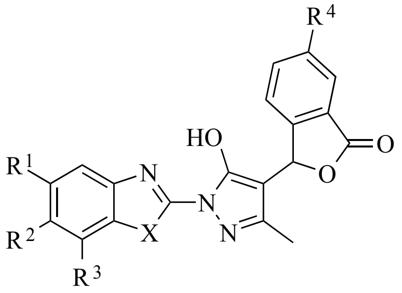

	R^1	R^2	R^3	X	R^4	IC_{50} (μM)
RU166365 "RU.365"	H	H	H	NH	H	1.89
RU320521	H	Cl	Cl	NH	H	0.11
RU320582	Cl	H	Cl	NH	H	0.12
RU320467	H	H	Br	NH	H	0.38
RU320520	H	H	Cl	NH	H	0.62
RU320462	H	Br	H	NH	H	0.64
RU320461	H	Me	H	NH	H	0.86
RU320519	H	H	Me	NH	H	1.16
RU320469	H	H	H	NH	Br	1.32
RU320468	H	H	H	NH	OMe	1.43
RU281332	H	H	H	S	H	1.79

Supplementary Table 4. Commercially available and in house synthesized analogs of RU166365 tested for m-cGAS inhibition

Molecule identifier	CXSMILES	Vendor	IC_{50} (μM)
RU320521	CC1=NN(C2=NC3=CC=C(Cl)C(Cl)=C3N2)C(O)=C1C1OC(=O)C2=CC=CC=C12 |c:12,18,27,t:1,4,6,8,25,29|	In house synthesized	0.11
RU320582	CC1=NN(C2=NC3=CC(Cl)=CC(Cl)=C3N2)C(O)=C1C1OC(=O)C2=CC=CC=C12 |c:9,12,18,27,t:1,4,6,25,29|	In house synthesized	0.12
RU320467	CC1=NN(C2=NC3=CC=CC(Br)=C3N2)C(O)=C1C1OC(=O)C2=CC=CC=C12 |c:8,11,17,26,t:1,4,6,24,28|	In house synthesized	0.38
RU320520	CC1=NN(C2=NC3=CC=CC(Cl)=C3N2)C(O)=C1C1OC(=O)C2=CC=CC=C12 |c:8,11,17,26,t:1,4,6,24,28|	In house synthesized	0.62
RU320462	CC1=NN(C2=NC3=CC=C(Br)C=C3N2)C(O)=C1C1OC(=O)C2=CC=CC=C12 |c:11,17,26,t:1,4,6,8,24,28|	In house synthesized	0.64
RU320461	CC1=NN(C2=NC3=CC=C(C)C=C3N2)C(O)=C1C1OC(=O)C2=CC=CC=C12 |c:11,17,26,t:1,4,6,8,24,28|	In house synthesized	0.86
RU320519	CC1=NN(C2=NC3=CC=CC(C)=C3N2)C(O)=C1C1OC(=O)C2=CC=CC=C12 |c:8,11,17,26,t:1,4,6,24,28|	In house synthesized	1.16
RU320469	CC1=NN(C2=NC3=CC=CC=C3N2)C(O)=C1C1OC(=O)C2=CC(Br)=CC=C12 |c:8,10,16,26,t:1,4,6,23,28|	In house synthesized	1.32
RU320468	COC1=CC=C2C(OC(=O)C2=C1)C1=C(O)N(N=C1C)C1=NC2=CC=CC=C2N1 |c:11,14,18,26,28,t:2,4,22,24|	In house synthesized	1.43
RU281332	CC1=NN(C2=NC3=CC=CC=C3S2)C(O)=C1C1OC(=O)C2=CC=CC=C12 |c:8,10,16,25,t:1,4,6,23,27|	Vitas-M Laboratory, Ltd.	1.79
**RU166365**	**CC1=NN(C2=NC3=CC=CC=C3N2)C(O)=C1C1OC(=O)C2=C1C=CC=C2 |c:8,10,16,23,26,28,t:1,4,6|**	**ChemBridge**	**1.89**
RU320464	CC1=NN(C2=NC3=CC=CC=C3N2)C(O)=C1C1OC(=O)C2=CC=C(Br)C=C12 |c:8,10,16,t:1,4,6,23,25,28|	In house synthesized	2.24
RU320575	CC1=NN(C2=NC3=CC=CC(=C3N2)C2=CC=CC=C2)C(O)=C1C1OC(=O)C2=CC=CC=C12 |c:8,10,17,19,23,32,t:1,4,6,15,30,34|	In house synthesized	2.35
RU320579	COC(=O)CC1=CC=CC2=C1C(=O)OC2C1=C(O)N(N=C1C)C1=NC2=CC=CC=C2N1 |c:7,9,17,21,29,31,t:5,25,27|	In house synthesized	2.46
RU320463	CC1=NN(C2=NC3=CC=CC=C3N2)C(O)=C1C1OC(=O)C2=C1C=CC=C2Br |c:8,10,16,23,26,28,t:1,4,6|	In house synthesized	2.76
RU320581	CC1=NN(C2=NC3=CC=CC(=C3N2)C(F)(F)F)C(O)=C1C1OC(=O)C2=CC=CC=C12 |c:8,10,20,29,t:1,4,6,27,31|	In house synthesized	3.30
RU320578	CC1=NN(C2=NC3=CC=C(C=C3N2)C2=CC=CC=C2)C(O)=C1C1OC(=O)C2=CC=CC=C12 |c:8,10,17,19,23,32,t:1,4,6,15,30,34|	In house synthesized	3.36
RU320460	COC1=C2NC(=NC2=CC=C1)N1N=C(C)C(C2OC(=O)C3=CC=CC=C23)=C1O |c:2,5,8,10,24,29,t:14,22,26|	In house synthesized	5.61
RU320511	COC(=O)C1=CC=CC2=C1C(=O)OC2C1=C(O)N(N=C1C)C1=NC2=CC=CC=C2N1 |c:6,8,16,20,28,30,t:4,24,26|	In house synthesized	6.84
RU320515	CC(C)C1=NN(C2=NC3=CC=CC=C3N2)C(O)=C1C1OC(=O)C2=CC=CC=C12 |c:10,12,18,27,t:3,6,8,25,29|	In house synthesized	7.16

RU320459	COC1=CC=C2N=C(NC2=C1)N1N=C(C)C(C2OC(=O)C3=CC=CC=C23)=C1O |c:6,10,24,29,t:2,4,14,22,26|	In house synthesized	7.34
RU320512	COC(=O)C1=CC=C2C(=O)OC(C2=C1)C1=C(O)N(N=C1C)C1=NC2=CC=CC=C2N1 |c:13,16,20,28,30,t:4,6,24,26|	In house synthesized	8.58
RU320502	CC1=NN(C2=NC3=NC=CC=C3N2)C(O)=C1C1OC(=O)C2=CC=CC=C12 |c:8,10,16,25,t:1,4,6,23,27|	In house synthesized	11.01
RU320577	CC1=NN(C2=NC3=CC=C(C=C3N2)C(O)=O)C(O)=C1C1OC(=O)C2=CC=CC=C12 |c:8,10,19,28,t:1,4,6,26,30|	In house synthesized	18.04
RU320504	CC1=NN(C2=NC3=CC=CC=C3N2)C(O)=C1C1OC(=O)C2=CC=CC(Br)=C12 |c:8,10,16,25,t:1,4,6,23,28|	In house synthesized	19.63
RU166418	CCCC1=C(O)N(N=C1C)C1=NC2=CC=CC=C2N1 |c:3,7,15,17,t:11,13|	Vitas-M Laboratory, Ltd.	>25
RU281319	CC1=CC=C(C=C1)C1=NN(C(O)=C1)C1=NC2=CC=CC=C2N1 |c:3,5,12,19,21,t:1,8,15,17|	InterBioScreen Ltd.	>25
RU281324	CC1=NN(C(O)=C1)C1=NC2=CC=CC=C2N1 |c:5,12,14,t:1,8,10|	Vitas-M Laboratory, Ltd.	>25
RU320465	CC1=NN(C2=NC3=CC=CC=C3N2)C(O)=C1C1NC(=O)C2=CC=CC=C12 |c:8,10,16,25,t:1,4,6,23,27|	In house synthesized	>25
RU320466	CC1=NN(C2=NC3=CC=CC=C3S2)C(O)=C1C1NC(=O)C2=CC=CC=C12 |c:8,10,16,25,t:1,4,6,23,27|	In house synthesized	>25
RU320503	COC1=CC=CC2=C1C(OC2=O)C1=C(O)N(N=C1C)C1=NC2=CC=CC=C2N1 |c:4,6,14,18,26,28,t:2,22,24|	In house synthesized	>25
RU320513	COC(=O)C1=CC=CC2=C1C(OC2=O)C1=C(O)N(N=C1C)C1=NC2=CC=CC=C2N1 |c:6,8,16,20,28,30,t:4,24,26|	In house synthesized	>25
RU320514	CC(C)C1=C(C2OC(=O)C3=CC=CC=C23)C(O)=NN1C1=NC2=CC=CC=C2N1 |c:3,11,18,26,28,t:9,13,22,24|	In house synthesized	>25
RU320516	CC1=NN(C2=NC3=CC=CC=C3N2)C(O)=C1C1OC(=O)C2=CC=CC(C(O)=O)=C12 |c:8,10,16,25,t:1,4,6,23,30|	In house synthesized	>25
RU320517	CC(=O)N1CCC2=C(C1C1=CC=CC=C1)C(C)=NN2C1=NC2=CC=CC=C2N1 |c:6,12,14,18,26,28,t:10,22,24|	In house synthesized	>25
RU320518	CC(=O)N1CCC2=NN(C3=NC4=CC=CC=C4N3)C(C)=C2C1C1=CC=CC=C1 |c:13,15,21,28,30,t:6,9,11,26|	In house synthesized	>25
RU320574	CC1=NN(C2=NC=C(N2)C2=CC=CC=C2)C(O)=C1C1OC(=O)C2=CC=CC=C12 |c:6,12,14,18,27,t:1,4,10,25,29|	In house synthesized	>25

.

Supplementary Table 5. Nucleic acids used in this study

Sequences used for dsDNA and hairpin RNA	
dsDNA	
45.50 (top)	5'-TACAGATCTACTAGTGATCTATGACTGATCTGTACATGATCTACA
45.52 (bottom)	5'-TGTAGATCATGTACAGATCAGTCATAGATCACTAGTAGATCTGTA
crystal (top)	5'-AAATTGCCGAAGACGAA
crystal (bottom)	5'-TTTCGTCTTCGGCAATT
hairpin RNA	
(HP20) 5'-ppp20L	5'ppp-GGAUCGAUCGAUCGAUCGGCUUCGGCCGAUCGAUCGAUCGAUCC-3'
Primer sequences used for qRT-PCR	
Actb1	
F	5'-CCCTAAGGCCAACCGTGAAAAG
R	5'-AGAGGCATACAGGGACAGCA
Il-6	
F	5'-CTTCACAAGTCGGAGGCTTAA
R	5'-ACTCCAGGTAGCTATGGTACTC
Ifnb1	
F	5'-GAGTTACACTGCCTTTGCCATCC
R	5'-ACTGTCTGCTGGTGGAGTTCAT
Primer sequences used for cloning of recombinant cGAS for use in crystal studies	
F	5'-GACGGATCCCCGGACAAGCTAAAGAAGGTGC
R	5'-GATGCGGCCGCTCAAAGCTTGTCAAAAATTGG
